# *Ureaplasma parvum* genotype, combined vaginal colonisation with *Candida albicans*, and spontaneous preterm birth in an Australian cohort of pregnant women

**DOI:** 10.1186/s12884-016-1110-x

**Published:** 2016-10-18

**Authors:** Matthew S. Payne, Demelza J. Ireland, Rory Watts, Elizabeth A. Nathan, Lucy L. Furfaro, Matthew W. Kemp, Jeffrey A. Keelan, John P. Newnham

**Affiliations:** 1 School of Women’s and Infants’ Health, University of Western Australia, 2nd Floor, Block A, King Edward Memorial Hospital, Subiaco, WA 6008 Australia; 2 Women and Infants Research Foundation, Biostatistics and Research Design Unit, Subiaco, WA 6008 Australia

**Keywords:** *Ureaplasma* spp, *Mycoplasma* spp, *Candida* spp, Genotyping, Vagina, Preterm birth

## Abstract

**Background:**

Detection of *Ureaplasma*, *Mycoplasma* and *Candida* spp. in the vagina during pregnancy has previously been associated with preterm birth (PTB). However, the prevalence of these microorganisms and the associated obstetric risks (likely to be population-specific) have not been determined in Australian women; furthermore, in the case of *Ureaplasma* spp., very few studies have attempted characterisation at the species level and none have examined genotype/serovar status to further refine risk assessment.

**Methods:**

In order to address these issues we sampled the vaginal fluid of 191 pregnant Australian women at three time points in pregnancy. Culture methods were used for detection of *Ureaplasma* spp. and *Candida* spp., and real-time PCR was used for speciation of *U. parvum* and *U. urealyticum*, non-albicans *Candida* spp., *Mycoplasma hominis* and *Mycoplasma genitalium*. High-resolution melt PCR was used to genotype *U. parvum*. Data on various lifestyle factors (including sex during pregnancy and smoking), antimicrobial use and pregnancy outcome were collected on all participants. Chi-square tests were used to assess the association of vaginal microorganisms with PTB.

**Results:**

Detection of *Ureaplasma* spp. was higher among spontaneous PTB cases, specifically in the presence of *U. parvum* [77 % preterm (95 % confidence interval (CI) 50–100 %) vs. 36 % term (CI: 29–43 %), *p* = 0.004], but not *U. urealyticum*. The association with PTB strengthened when *U. parvum* genotype SV6 was detected (54 % preterm (CI: 22–85 %) vs. 15 % term (CI: 10–20 %), *p* = 0.002); this genotype was also present in 80 % (4/5) of cases of PTB <34 weeks gestation. When present with *Candida albicans* in the same sample, the association with PTB remained strong for both *U. parvum* [46 % preterm (CI: 15–78 %) vs. 13 % term (CI: 8–18 %), *p* = 0.005] and *U. parvum* genotype SV6 [39 % preterm (CI: 8–69 %) vs. 7 % term (CI: 3–11 %), *p* = 0.003]. With the exception of *Candida glabrata*, vaginal colonisation status for all organisms was stable throughout pregnancy. Smoking significantly increased the likelihood of detection of all target organisms.

**Conclusions:**

These data suggest that the presence of different species and serovars of *Ureaplasma* spp. in the vagina confers an increased risk of spontaneous PTB, findings which may be useful in risk assessment for identifying women who would benefit from antimicrobial treatment.

## Background

The role of human genital Ureaplasmas (*Ureaplasma parvum* and *Ureaplasma urealyticum*) and Mycoplasmas (*Mycoplasma hominis* and *Mycoplasma genitalium*) in preterm birth (PTB) has been a contentious topic that has resulted in considerable research over the past two decades. A recent review article by Capoccia et al. [1], summarizing the bulk of this research effort (with the exception of *M. genitalium*), showed that the majority of studies with defined case/control subjects found an association with negative pregnancy outcomes, especially for *Ureaplasma* spp. For *M. hominis*, this association appeared to be increased when detected alongside *Ureaplasma* spp. [1]. *M. genitalium*, however, is more commonly associated with pelvic inflammatory disease [2] and has also been linked to bacterial vaginosis (BV) [3]. Vaginal colonisation by *Candida* spp., a common yeast synonymous with vaginal thrush, has also been associated with PTB risk [4–7].

However, it is still unclear why some women who are asymptomatically colonised with these organisms have pregnancies that deliver preterm while others deliver at term; possible explanations include interactions with other microorganisms and inflammatory modulators, the timing and duration of colonisation, differences in virulence between species/strains, and eradication or exacerbation via maternal immune responses. With respect to *Ureaplasma* spp., very few studies have ever attempted to refine the data beyond the genus level, despite evidence that numerous other Ureaplasma-associated disease phenotypes are differentially associated with *U. urealyticum* and not *U. parvum* [8–12]. Further to this, to the best of our knowledge, no studies have ever looked at the relationship that may exist between PTB and the presence of specific *Ureaplasma* spp. genotypes (serovars). In addition, few studies have looked at the effect of combined colonisation with these organisms, with most studies instead focusing on either *Ureaplasma* spp. and *M. hominis* (and not *M. genitalium*) or *Candida* spp.

PTB is now recognised as the leading cause of death in children under the age of 5 years in the developed world [13], with intrauterine infection being causally associated with ~40 % of all PTBs [14]. In particular, the births of the earliest gestational ages, which are associated with the greatest mortality and morbidity, are much more likely to be the consequence of intrauterine infection and/or inflammation (up to 80 %). There is, therefore, a need to be able to predict which women are at a high risk of PTB, based on factors other than previous clinical history alone, so that appropriate treatment may be provided to those who would benefit. This is especially relevant for women in their first pregnancy. Despite the body of research that has been conducted into the presence and significance of genital *Ureaplasma*, *Mycoplasma* and *Candida* spp. colonisation during pregnancy, there is very little information available on the relationship between specific lifestyle practices, such as smoking and sexual activity, and vaginal colonisation. However, it has been well established that tobacco use increases susceptibility to bacterial infection [15] and a strong association has been documented between smoking and BV [16–19]. Furthermore, some genital *Ureaplasma* and *Mycoplasma* spp. are deemed to be sexually transmitted infections [20].

In addition, the vast majority of previous studies that have looked at genital *Ureaplasma*, *Mycoplasma* and *Candida* spp., have focused on collection of clinical samples at a single time point. This approach provides no data on the colonisation dynamics of a particular organism and this is likely to be of significance in terms of implementing diagnostic assays for the detection of women at risk of PTB. It is highly likely that all of the above information combined, will not only increase our understanding of these organisms during pregnancy, but importantly, will allow us to establish useful microbial biomarkers. These, combined with patient-specific qualitative data, will improve our ability to predict women at an increased risk of PTB who would benefit from anti-microbial treatment. Due to previous descriptions of variation in the vaginal microbiota as a result of ethnic, cultural and social factors [21], it is likely that microbial colonisation status and PTB risk are strongly population-dependent.

The aims of this study, therefore, were to document the presence of *U. parvum* (including genotype analysis), *U. urealyticum*, *C. albicans*, *C. glabrata*, other non-albicans *Candida* spp., *M. hominis* and *M. genitalium* in the vagina of a cohort of asymptomatic pregnant Australian women at three time points during pregnancy, and to examine any association between their microbiological characteristics, a range of lifestyle factors, and risk of PTB.

## Methods

### Subjects

The study consisted of 206 low-risk pregnant women recruited from King Edward Memorial Hospital (KEMH), Perth, Western Australia. Fifteen cases withdrew from the study or were lost to follow-up, leaving 191 for analysis. The study was approved by the Human Research Ethics Committee of the Western Australian Department of Health, Women and Newborn Health Service (2056/EW).

### Inclusion and exclusion criteria

Women with a singleton pregnancy were eligible for inclusion if they were aged 18–40 years, able to speak and read English and were pregnant within the first or second trimester.

Women were excluded from the study if they were deemed to be at a high risk of PTB (one or more previous PTBs) and/or other pregnancy complications such as preeclampsia. Other exclusion criteria included current use of antifungals, tetracycline and/or macrolide antibiotics, current diagnosis of a urinary tract infection, and history of recurrent vaginal thrush.

### Questionnaires

Upon recruitment to the study and at each subsequent sampling point, women were invited to complete a de-identified medical/lifestyle questionnaire in a private setting. The questionnaire first inquired about medications currently used (antibiotic/natural/probiotic) and past diagnoses of urinary tract infections/vaginal thrush. Information regarding current and previous smoking and/or alcohol use was sought as ‘yes or no’, and subsequently followed by questions to quantify the number of cigarettes smoked/standard drinks consumed each day as appropriate. The average number of episodes of sexual intercourse per week during pregnancy was recorded.

### Pregnancy outcome data

Pregnancy outcome data from the hospital’s electronic medical records were accessed by experienced research midwives and coded after completion of the pregnancy.

### Sample collection

Written informed consent was obtained by the attending midwife prior to enrolment in the study. This included consenting to publication of any data produced by the study. Participants provided two self-collected vaginal swabs (Copan Diagnostics, Murrieta, CA, USA) at recruitment (median 21 week, range 13–26 weeks gestational age [GA]), ~28 weeks GA (median 29, range 24–38 weeks) and ~36 weeks GA (median 36, range 32–40 week). The first swab was employed for detection of *Ureaplasma* and *Mycoplasma* spp. and the second for detection of *Candida* spp. Detailed verbal, written and pictorial instructions were provided to all women in an attempt to standardise the swab collection process. Briefly, while wearing gloves, participants inserted the swab 5 cm into their vagina and gently rotated this for 20 s, ensuring the walls of the vagina came into contact with the swab. Swabs were then immediately placed into a collection tube containing either 1 mL UTM media (*Ureaplasma* and *Mycoplasma* spp.) (Copan Diagnostics) or 2 mL of CAT media (*Candida* spp.) (Copan Diagnostics), snapped at the mid-stem breakpoint, capped and stored at 4 °C. All samples were transported to the laboratory on ice for culture within 24 h of collection.

### Detection of *Ureaplasma* spp.

#### Culture

UTM tubes were vortexed for 10 s to release all cells from swabs. Swabs were subsequently pressed against the tube wall to release all free liquid and then discarded. Two hundred microliters of sample was added to 1.8 mL of 10B broth (Melbourne University Media Preparation Unit) and incubated for 48 h at 37 °C, 5 % CO_2_, 2 % O_2_. The remaining volume of sample was transferred to a 2 mL microfuge tube and frozen at −80 °C until DNA extraction.

Positive cultures, indicated by a pH-associated colour change (yellow > pink), were immediately transferred to 2 mL microfuge tubes and frozen at −80 °C.

#### DNA extraction

DNA was extracted from 250 μL of UTM swab eluate using the Siemens Sample Preparation Kit 1.0 (Siemens, Munich, Germany) on an automated Kingfisher Duo extraction platform (Thermo Fisher Scientific Inc. MA, USA) as per manufacturer’s instructions. All extracts were eluted in a final volume of 100 μL of elution buffer (Siemens). A positive extraction control consisting of approximately 250 colour changing units (CCU) each of *U. parvum* and *U. urealyticum* was included in all runs.

#### Real-time PCR

In addition to culture, *Ureaplasma* spp. DNA was detected from vaginal swabs using real-time PCR. Vaginal swab DNA was screened using an assay targeting the urease gene of *U. parvum* and *U. urealyticum*, as described by Yi et al. [22], adapted for use on a ViiA7 real-time PCR system (Life Technologies, Carlsbad, CA, USA). Reaction mixtures (final concentration) consisted of 1X Taqman FAST Advanced Master Mix (Life Technologies), 0.9 μM primers UU1613F and UU1524R (Life Technologies), 0.25 μM probes UU-parvo (FAM) and UU-T960 (VIC) (Life Technologies), 5 μL of template DNA and nuclease-free water (Ambion, Life Technologies) to a final volume of 20 μL. PCR cycling conditions consisted of an initial denaturation/Taq activation at 95 °C for 20 s, followed by 40 quantification cycles of 95 °C for 1 s and 60 °C for 20 s (data acquiring). Positive standards were included in each run.

#### High-resolution melt PCR

Samples that were positive for *U. parvum* DNA were genotyped and classified as either serovar (SV) one, SV3, SV6 or SV14 using our previously described high resolution melt (HRM) PCR assay targeting the multiple-banded antigen gene [23] on a ViiA7 real-time PCR system (Life Technologies). Reaction mixtures (final concentration) consisted of 1X Amplitaq Gold 360 buffer (Life Technologies), 1.5 mM MgCl_2_ (Life Technologies), 200 μM of each dNTP (Life Technologies), 0.3 μM primers UPHRM-F and UPHRM-R (Life Technologies), 1X MeltDoctor HRM dye (Life Technologies), Amplitaq Gold 360 DNA polymerase (0.1 U/μL) (Life Technologies), 10 μL of template DNA and nuclease-free water (Ambion, Life Technologies) to a final volume of 20 μL. PCR cycling conditions consisted of an initial denaturation/Taq activation at 95 °C for 10 min, followed by 40 cycles of 95 °C for 15 s and 60 °C for 1 min (data acquiring). To provide data on *U. parvum* serovar status, amplicons were subsequently subject to a HRM step where the temperature was raised to 95 °C for 10 s and then lowered to 60 °C for 1 min. The temperature was then raised to 95 °C at a rate of 0.025 °C/s (continuous data acquisition), held at 95 °C for 15 s and then lowered to 60 °C for 15 s. HRM profiles were analysed using ViiA7 real-time PCR system software v1.2.1 (Life Technologies). All samples were run in duplicate and positive standards of *U. parvum* SV1, SV3, SV6 and SV14 were included in each run.

#### Sequencing

Following HRM analysis, samples that produced non-standard melt curve patterns were subject to DNA sequencing. PCR amplicons were generated using the same HRM primer set on a Veriti PCR thermocycler (Life Technologies). Reaction mixtures (final concentration) consisted of 1X Amplitaq Gold 360 buffer (Life Technologies), 2.0 mM MgCl_2_ (Life Technologies), 200 μM of each dNTP (Life Technologies), 0.5 μM primers UPHRM-F and UPHRM-R (Life Technologies), Amplitaq Gold 360 DNA polymerase (1.25U) (Life Technologies), 5 μL of template DNA and nuclease-free water (Ambion, Life Technologies) to a final volume of 50 μL. PCR cycling conditions consisted of an initial denaturation/Taq activation at 95 °C for 10 min, followed by 40 cycles of 95 °C for 15 s, 56 °C for 30 s and 72 °C for 45 s. A final extension step of 72 °C for 7 min was also included.

PCR amplicons were checked for size (305 bp) on a 1.5 % agarose gel stained with Gel Red (Biotium) and subsequently purified using a QIAquick PCR purification kit (QIAGEN) as per manufacturer’s instructions. Purified DNA fragments were sequenced using Big Dye version 3.1 chemistry (Applied Biosystems) and post-cleaned using SPRI. Fragments were separated on a 3730xI DNA Analyser using a 96-capillary array (Applied Biosystems) at the Australian Genome Research Facility (Perth, Western Australia).

### Detection of *Mycoplasma* spp.

#### DNA extraction

DNA was extracted from 250 μL of UTM swab eluate as described above.

#### Real-time PCR

##### Mycoplasma hominis

*M. hominis* DNA was detected in vaginal swabs using real-time PCR. DNA samples were screened using an assay targeting the *yidC* gene of *M. hominis* as described by Ferandon et al. [24], adapted for use on a ViiA7 real-time PCR system (Life Technologies). Reaction mixtures (final concentration) consisted of 1X Taqman FAST Advanced Master Mix (Life Technologies), 0.9 μM primers MHyidCfwd and MHyidCrev (Life Technologies), 0.25 μM probe MHyidC (FAM) (Life Technologies), 5 μL of template DNA and nuclease-free water (Ambion, Life Technologies) to a final volume of 20 μL. PCR cycling conditions were as described for *Ureaplasma* spp. A positive standard was included in each run.

##### Mycoplasma genitalium

*M. genitalium* DNA was detected in vaginal swabs using real-time PCR. DNA samples were screened using an assay targeting the *MgPa* gene of *M. genitalium* as described by Jensen et al. [25], adapted for use on a ViiA7 real-time PCR system (Life Technologies). Reaction mixtures (final concentration) consisted of 1X Taqman FAST Advanced Master Mix (Life Technologies), 0.9 μM primers MgPa-355F and MgPa-432R (Life Technologies), 0.25 μM probe MgPa-380 (VIC) (Life Technologies), 7.9 μL of template DNA and nuclease-free water (Ambion, Life Technologies) to a final volume of 20 μL. PCR cycling conditions consisted of an initial denaturation/Taq activation at 95 °C for 20 s, followed by 50 quantification cycles of 95 °C for 1 s and 60 °C for 20 s (data acquiring). Positive standards were included in each run.

### Detection of *Candida* spp.

#### Culture

CAT tubes were vortexed for 10 s to release all cells from swabs. Swabs were subsequently pressed against the tube wall to release all free liquid and then disposed of. One millilter of sample was transferred to a 2 mL microfuge tube and frozen at −80 °C until DNA extraction. The remaining sample (approximately 900 μL) was incubated at 37 °C for 24 h to enrich for low cell titres of *Candida* spp. Following incubation, two 10 μL loops of sample were plated onto Candida Brilliance agar (Oxoid, Thebarton, South Australia, Australia) and incubated at 37 °C for 72 h.

Positive cultures on Candida Brilliance agar (Oxoid) were classified as follows: Green colonies = *C. albicans*; pink/yellow/beige/brown colonies = non-albicans *Candida* spp. All positive cultures were re-plated for purity and following incubation, pure cultures were re-suspended in 2 mL of Sabaraud-Dextrose broth (Oxoid) and frozen at −80 °C.

#### DNA extraction

DNA was extracted from 250 μL of pure *Candida* sp. isolate broth resuspension as described above.

#### Real-time PCR

To confirm the identification of non-albicans *Candida* spp. isolated using Candida Brilliance agar, a multiplex real-time PCR assay targeting the RNase P RNA (RPR) gene of *Candida* sp. and *C. glabrata* was used. Primer and probe designs were similar to that of Innings et al. [26], but were optimised for use on a ViiA7 real-time PCR system (Life Technologies). Reaction mixtures (final concentration) consisted of 1X Taqman FAST Advanced Master Mix (Life Technologies), 0.9 μM primers CAND-CR1F (5′ CGGGTGGGAAATTCGGT 3′), CAND-CR5R (5′ CAATGATCGGTATCGGGT 3′), GLA-F (5′ TGGCTCACACACTTTGTCACTTT 3′) and GLAR (5′ ACCTCGCCTCACACCAATG 3′) (Life Technologies), 0.25 μM probes ALLCAN (NED-TTCGCATATTGCACTMAAYAGC-MGB) and GLA (VIC-AACCTGCCATTTCCGCTCCCTTAAGA-TAMRA) (Life Technologies), 5 μL of template DNA and nuclease-free water (Ambion, Life Technologies) to a final volume of 20 μL. PCR cycling conditions were as described above for *Ureaplasma* spp.

### Statistical analyses

Data were summarised using frequency distributions for categorical data, and median, interquartile range and range for continuous data. Categorical outcomes were compared using Chi-square and Fisher’s exact tests, and continuous outcomes compared using Mann-Whitney tests. All analyses were conducted on detection of microbes at recruitment, due to the relatively stable colonisation levels throughout pregnancy. SPSS Version 20.0 (Armonk, NY: IBM Corp) statistical software was used for data analysis. *P*-values <0.05 were considered statistically significant.

## Results

### Subjects

206 women in total were recruited to the study. From these, 15 withdrew or were lost to follow-up. Demographic/birth and lifestyle characteristics of the 191 women that formed the final study cohort are provided below (Table 1). The overall PTB rate (<37 weeks GA) was 9 %, which included 13 spontaneous births and four births that required labour induction or caesarean delivery for maternal or fetal indications. These four births were excluded in the comparison of microorganisms between preterm and term births. There were six births (five spontaneous) <34 weeks GA (3 %) and two births with a birth weight <1500 g (1 %).

**Table 1:** Demographic, birth and lifestyle characteristics of women in the study^a^

Demographic and birth	*n* = 191
Maternal age (y)	30 (26–33;18–43)
Caucasian	119 (62 %)
GA at birth	39 (38–40; 26–42)
GA <37 weeks	17 (9 %)
Birthweight (g)	3435 (3080–3733;690–4794)
Gender male	92 (48 %)
Lifestyle and medication	*n* = 189
Current antibiotic, antifungal or probiotic medication	8 (4 %)
UTI diagnosis ever	75 (40 %)
Thrush diagnosis ever	91 (48 %)
Thrush > 2 times	12 (13 %)
Current smoker	21 (11 %)
Smoke >10/day	5 (3 %)
Previous smoker	50 (27 %)
Smoke >10–20/day	13 (7 %)
Smoke >20/day	5 (3 %)
Current alcohol	4 (2 %)
≥ 5 drinks/week	1 (0.5 %)
Previous alcohol	108 (57 %)
< 1 drink/week	17 (9 %)
1–4 drinks/week	64 (34 %)
≥ 5 drinks per wk	18 (9 %)
Current sexual intercourse	
< 1 week	42 (23 %)
1–2 times/week	96 (52 %)
≥ 3 times/week	48 (26 %)

*y* years, *GA* gestational age, *wk* weeks, *g* grams

^a^Data represents median (interquartile range; range) or *N* (%), as appropriate

### Detection of vaginal *Ureaplasma*, *Mycoplasma* and *Candida* spp. during pregnancy

Vaginal detection rates for *Ureaplasma* spp., *Mycoplasma* spp. and *Candida* spp. varied substantially at both the genus and species level (Table 2). *Ureaplasma* spp. were the most common of the three organisms detected, present in 44–48 % of women over the three sampling points. Within this genus, *U. parvum* was the most common species detected, 3–4 times more prevalent than *U. urealyticum* (Table 2).

**Table 2:** Detection rates for vaginal *Ureaplasma*, *Mycoplasma* and *Candida* spp. during pregnancy^a^

Organisms	Time-point 1 (*n* = 191)	Time-point 2 (*n* = 154)	Time-point 3 (*n* = 152)
Gestation at sampling (wk)^b^	21 (13–26)	29 (24–38)	36 (32–40)
*Ureaplasma* spp.	91 (48 %)	74 (48 %)	67 (44 %)
*U. parvum*	74 (39 %)	63 (41 %)	53 (35 %)
SV1	11 (5.8 %)	10 (6.5 %)	10 (6.6 %)
SV3	27 (14.1 %)	20 (13 %)	19 (12.5 %)
SV6	31 (16.2 %)	24 (15.6 %)	18 (11.8 %)
SV6.1	1 (0.5 %)	1 (0.6 %)	1 (0.7 %)
SV14	0 (0 %)	0 (0 %)	0 (0 %)
Mixed	2 (1 %)	2 (1.3 %)	3 (2 %)
Too weak to call	0 (0 %)	3 (1.9 %)	0 (0 %)
No amplification	2 (1 %)^c^	2 (1.3 %)^c^	2 (1.3 %)^c^
*U. urealyticum*	25 (13 %)	15 (10 %)	16 (11 %)
*Candida* spp.	73 (38 %)	53 (34 %)	52 (34 %)
*C. albicans*	63 (33 %)	50 (33 %)	48 (32 %)
*C. glabrata*	10 (5 %)	2 (1 %)	3 (2 %)
Non-albicans/non-glabrata *Candida* spp.	5 (3 %)	5 (3 %)	2 (1 %)
*M. hominis*	21 (11 %)	12 (8 %)	16 (11 %)
*M. genitalium*	6 (3 %)	3 (2 %)	4 (3 %)

^a^Due to variations in sample compliance, apparent reductions or increases in genotypes over the three time points are not indicative of genotype stability

^b^represents median, range

^c^Same study participants

*Candida* spp. were the second most common organism detected, present in 34–38 % of women. Within this genus, *C. albicans* was by far the most common species detected, 6–25 and 10–24 times more prevalent than *C. glabrata* and non-albicans/non-glabrata *Candida* spp., respectively (Table 2).

Detection rates for *M. hominis* and *M. genitalium* were much lower than for *Ureaplasma* spp. and *Candida* spp. *M. hominis* detection rates ranged from 8 to 11 % over the three sampling points, whilst for *M. genitalium*, rates ranged from 2 to 3 % (Table 2).

### Comparison of culture and real-time PCR for detection of *Ureaplasma* spp.

Detection rates of *Ureaplasma* spp. by 10B broth culture at 37 °C (5 % CO_2_, 95 % N_2_) were almost identical to detection rates by real-time PCR. Concordance between the two techniques was 99, 100 and 100 % over the three time-points, respectively.

### High-resolution melt PCR genotyping of *U. parvum*

HRM PCR was able to resolve singular *U. parvum* genotypes in 91 % of colonised clinical samples. *U. parvum* genotype SV6 was the most common detected, closely followed by SV3, SV1 and SV6.1, respectively. No cases of genotype SV14 were found (Table 2). An additional 3 % of cases were resolved to the ‘mixed’ genotype level, suggesting the presence of two or more *U. parvum* genotypes within the same sample. There was also a small number of cases where either amplification was too weak to produce a melt curve sufficient for genotype discrimination (1 %) or no amplification was produced whatsoever (3 %). In addition, for one study participant, all three samples produced slightly different HRM curves, which upon DNA sequencing, showed four unique nucleotide polymorphisms within the targeted region of the multiple-banded antigen gene, not indicative of any of the four characterised *U. parvum* genotypes. The sequence was most closely matched to genotype SV6, and as a result was deemed genotype SV6.1.

### Vaginal colonisation dynamics

**Fig. 1:**
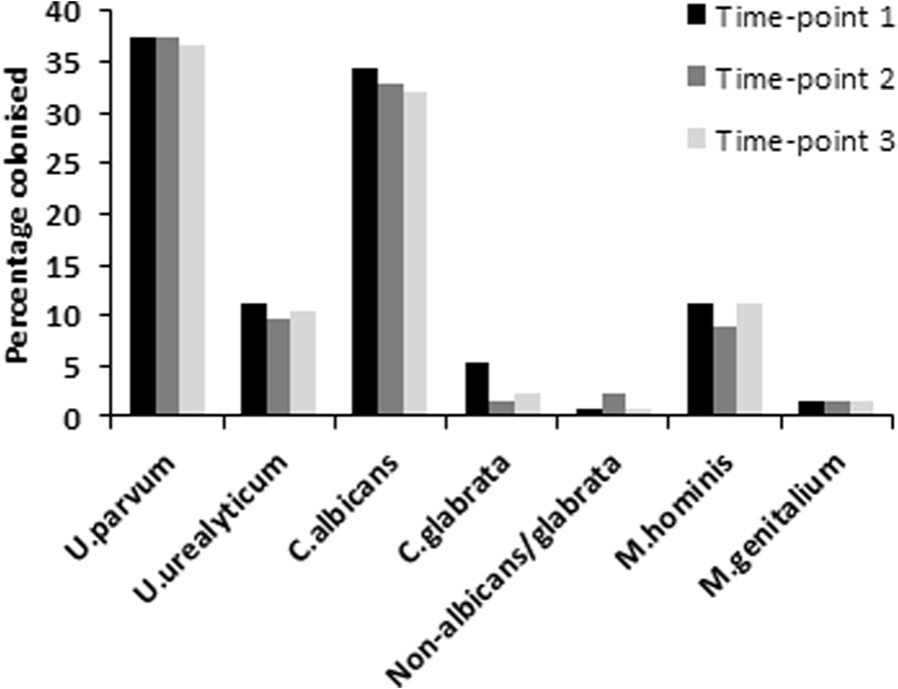
Prevalence of *Ureaplasma*, *Candida* and *Mycoplasma* spp. in vaginal swab samples from 134 women with three completed samples taken over the course of the pregnancy. *Solid black, sample time-point 1; dark grey, sample time-point 2; light grey, sample time-point 3*

Of the 191 study participants, 134 provided samples at all three time points. In these women, detection rates for all organisms showed minimal variance over the duration of the study (Fig. 1). *M. genitalium* (1.5 % at all three points) had the lowest variance of all organisms detected, followed by *U. parvum* (36.6–37.3 %), *U. urealyticum* (9.7–11.2 %) and non-albicans/non-glabrata *Candida* spp. (0.7–2.2 %), *C. albicans* (32.1–34.3 %) and *M. hominis* (9–11.2 %), and last, *C. glabrata* (1.5–5.2 %). These results are inclusive of one case where *U. parvum*/*U. urealyticum* was detected at recruitment sampling, after which *U. urealyticum* was not detected again in subsequent samples.

Similarly, there was very little variance in *U. parvum* genotype detection at any of the three time points. In nearly all cases, the genotype detected at recruitment was maintained throughout the second and third time points. The only exceptions were in one case where a participant colonised by *U. parvum* genotype SV6 at recruitment showed a mixed genotype profile at the later time points, and in another case where mixed genotype profiles were detected at all three time points. This made it impossible to be certain that the same combination of genotypes was present in each instance, due to the limitations of the HRM assay. There were also an additional two instances where the HRM assay failed to generate sufficient amplification of the second sample to allow accurate genotype discrimination. However, in both of these cases, the first and third sample genotype identifications were concordant.

### Association between pharmaceutical & lifestyle factors and detection of organisms at recruitment

Based upon answers provided by 189 study participants at recruitment (Table 3), *Ureaplasma* spp. and *Mycoplasma* spp. were detected more frequently in women who previously smoked (*Ureaplasma* spp. −37 % present vs. 17 % absent, *p* = 0.002; and *Mycoplasma* spp. −44 % present vs. 24 % absent, *p* = 0.036), and in women who had sexual intercourse ≥ 3 times per week during pregnancy (*Ureaplasma* spp. −35 % present vs. 18 % absent, *p* = 0.018; and *Mycoplasma* spp. −56 % present vs. 21 % absent, *p* = 0.001).

**Table 3:** Association between pharmaceutical/lifestyle characteristics and detection of *Ureaplasma*, *Candida*, and *Mycoplasma* spp. at recruitment (*n* = 189)^a^

Question	*Ureaplasma* spp.		*p*-value	*Candida* spp.		*p*-value	*Mycoplasma* spp.		*p*-value
	Yes (*n* = 89)	No (*n* = 100)		Yes (*n* = 73)	No (*n* = 116)		Yes (*n* = 25)	No (*n* = 164)	
Current antibiotic, antifungal or probiotic use?	**1 (1 %)**	**7 (7 %)**	**0.045**	3 (4 %)	5 (4 %)	0.947	1 (4 %)	7 (4 %)	1.000
Ever diagnosed with a UTI?	32 (36 %)	43 (43 %)	0.296	30 (41 %)	45 (39 %)	0.789	8 (32 %)	67 (41 %)	0.387
Ever diagnosed with thrush?	40 (46 %)	51 (51 %)	0.448	34 (47 %)	57 (49 %)	0.798	10 (42 %)	81 (49 %)	0.479
Current smoker?	13 (15 %)	8 (8 %)	0.149	**13 (18 %)**	**8 (7 %)**	**0.020**	5 (20 %)	16 (10 %)	0.165
Previous smoker?	**33 (37 %)**	**17 (17 %)**	**0.002**	22 (31 %)	28 (24 %)	0.351	**11 (44 %)**	**39 (24 %)**	**0.036**
Currently consumes alcohol?	1 (1 %)	3 (3 %)	0.624	2 (3 %)	2 (2 %)	0.642	1 (4 %)	3 (2 %)	0.438
Previously consumed alcohol?	48 (54 %)	60 (61 %)	0.355	39 (53 %)	69 (60 %)	0.374	14 (56 %)	94 (58 %)	0.875
Current frequency of sexual intercourse?									
< 1 week	20 (23 %)	22 (23 %)	**0.018**	14 (20 %)	28 (24 %)	0.715	3 (12 %)	39 (24 %)	**0.001**
1–2 times/week	38 (43 %)	58 (60 %)		37 (52 %)	59 (51 %)		8 (32 %)	88 (55 %)	
≥ 3 times/week	**31 (35 %)**	**17 (18 %)**		20 (28 %)	28 (24 %)		**14 (56 %)**	**34 (21 %)**	

^a^ *wk* week; bold type indicates statistical significance (*p* < 0.05)

*Candida* spp. were detected more frequently in women who continued to smoke during their pregnancy (*Candida* spp. −18 % present vs. 7 % absent, *p* = 0.020).

### Association between vaginal microbial colonisation and spontaneous preterm birth

#### Spontaneous preterm birth <37 weeks GA

The overall microbial characteristics of vaginal samples collected during the study are provided in Table 4. *Ureaplasma* spp. were detected more frequently [85 % (95 % CI: 62–100 %) vs. 45 % (37–52 %), *p* = 0.006] in the recruitment samples of women who delivered preterm compared to those who delivered at term (Table 4). At the species level, the presence of *U. parvum* was significantly increased among PTB cases [77 % (50–100 %) vs. 36 % (29–43 %), *p* = 0.004]. There was a small, but significant association between the titre of *U. parvum* and PTB, with an average *U. parvum* titre of 10^6^ CCU in cases of PTB vs. 10^5^ CCU for term cases. *U. parvum* genotypes SV3 and SV6 were equally represented amongst term pregnancies; however, in women who delivered preterm, genotype SV6 was significantly more common, present in 54 % (22–85 %) of preterm deliveries compared to 15 % (10–20 %) of term deliveries (*p* = 0.002). When detected alone, the presence of *Candida* spp. was not associated with PTB at either the genus or species level. However, when *C. albicans* was detected alongside *U. parvum* a significant positive association with PTB was observed [46 % (15–78 %) vs. 13 % (8–18 %), *p* = 0.005]. This association strengthened when *U. parvum* genotype SV6 was present [39 % (8–69 %) vs. 7 % (3–11 %), *p* = 0.003].

**Table 4:** Vaginal colonisation rates of *Ureaplasma*, *Candida* and *Mycoplasma* spp. at recruitment in women who delivered spontaneously preterm vs. at term^a^

	Preterm *N* = 13	Term *N* = 174	*p*-value
***Ureaplasma*** **spp.**	**11 (85 %)**	**78 (45 %)**	**0.006**
***U. parvum***	**10 (77 %)**	**63 (36 %)**	**0.004**
*U. urealyticum*	2 (15 %)	23 (13 %)	0.687
**Titre (CCU) of** ***U*** **.** ***parvum*** ^b^	**6 (5–6;3–7)**	**5 (4–6;2–8)**	**0.043**
Serovar 3 of *U. parvum*	3 (23 %)	23 (13 %)	0.399
**Serovar 6 of** ***U. parvum***	**7 (54 %)**	**26 (15 %)**	**0.002**
*Candida* spp*.*	7 (54 %)	63 (36 %)	0.241
*C. albicans*	6 (46 %)	55 (32 %)	0.358
*C. glabrata*	1 (8 %)	9 (5 %)	0.522
*Non-albicans/non-glabrata*	0	4 (2 %)	1.000
*Mycoplasma* spp.			
*M. hominis*	2 (15 %)	19 (11 %)	0.644
*M. genitalium*	2 (15 %)	4 (2 %)	0.057
***U. parvum*** **+** ***C. albicans***	**6 (46 %)**	**22 (13 %)**	**0.005**
***U. parvum serovar*** **6 +** ***C. albicans***	**5 (39 %)**	**12 (7 %)**	**0.003**

^a^Bold type indicates statistical significance (*p* < 0.05)

^b^median, interquartile range, range

There was no apparent association between presence of *Mycoplasma* spp. and PTB; however, *M. genitalium* was more common in the recruitment samples from women who delivered preterm vs. at term (15 vs. 2 %, respectively) and this result trended towards significance (*p* = 0.057). Similarly, *Candida* spp. were also more common in the recruitment samples from women who delivered preterm vs. at term (54 vs. 36 %, respectively); however, this difference was not statistically significant (*p* = 0.241).

No association was detected between PTB and the presence of either *U. urealyticum*, *C. albicans*, *C. glabrata*, non-albicans/glabrata *Candida* spp. or *M. hominis*.

#### Spontaneous preterm birth <34 weeks GA and birthweight <1500 g

Five babies were born <34 weeks GA, including two weighing <1500 g (Table 5). *U. parvum* was detected at recruitment in all cases and in 80 % (4/5) of these, genotype SV6 was present. In the four earliest preterm deliveries (25.9–31.4 weeks GA), both *U. parvum* and *C. albicans* were detected at recruitment (Table 5). Unfortunately, due to insufficient numbers, no statistical analyses were able to be performed on risk of PTB <34 weeks GA.

**Table 5:** Detection of organisms at recruitment and birth characteristics for babies born spontaneously at <34 weeks GA (*n* = 5)^a^

GA (wk)	BW (g)	Sex	*U. parvum*	Titre (CCU – 10^^^)	*U. parvum/U. urealyticum*	*U. parvum* SV6	*C. albicans*	*M. hominis*	*M. genitalium*
25.9	690	M	✓	6	*U. parvum*	✓	✓	✓	✓
28.0	760	M	✓	7	*U. parvum*	✓	✓	-	-
30.9	1560	M	✓	6	*U. parvum*	-	✓	✓	-
31.4	1950	M	✓	5	Both	✓	✓	-	✓
32.0	1820	F	✓	6	*U. parvum*	✓	-	-	-

^a^ *wk* weeks, *g* grams, *CCU* colour changing units; a tick (✓) represents presence of organism, a dash (−) absence of organism

## Discussion

Numerous previous studies have described an association between detection of *Ureaplasma* spp. in the vagina during pregnancy and subsequent PTB [1]. Consistent with data from the majority of these studies, our findings confirm a significant association between vaginal colonisation by *Ureaplasma* spp. and PTB risk. However, unlike the vast majority of previous research, we have further discriminated between human Ureaplasmas at the species level. Our finding that vaginal colonisation by *U. parvum*, but not *U. urealyticum*, was associated with an increased risk of PTB, compared to colonisation by *Ureaplasma* spp. in general, highlights a major limitation in these previous studies. This is despite the fact that in 1990 *U. urealyticum* was classified as two distinct biovars, parvo (*U. parvum*) and T960 (*U. urealyticum*) [27], and then formally proposed as two separate species in 2002 [28]. In addition, species (biovar)-level PCR detection assays have been available since 1993 [29]; hence, the ability to study this organism at the species level has existed for many years. It is important that future studies ensure that species-level analyses are carried out, especially those examining pregnancy outcome, as our data showed that *U. parvum* confers the greatest risk of PTB. This is in stark contrast to other disease phenotypes, such as pelvic inflammatory disease [8], endometritis [8], non-gonococcal urethritis [8–10, 12] and post-gonococcal urethritis [11], where *U. urealyticum* has been reported to be significantly associated with disease, but not *U. parvum*. In previous studies [30–45] that examined the obstetric consequences of vaginal colonisation by *Ureaplasma* spp., where there were defined case and control groups, only three discriminated between *U. parvum* and *U. urealyticum*. Similar to our study, the two most recent of these, Mitsunari et al. [32] and Kataoka et al. [42] reported that vaginal colonisation by *U. parvum*, but not *U. urealyticum*, was significantly associated with PTB. However, in contrast, a study from the late 1990s by Abele-Horn et al. [34] came to the opposite conclusion, with *U. urealyticum* apparently having more adverse effects on birth weight, gestational age and preterm delivery compared to *U. parvum*. These are the only authors, to our knowledge, that have reported an association between *U. urealyticum* and PTB; however, they employed a unique set of inclusion criteria whereby women were recruited who were solely colonised with *Ureaplasma* spp. and no other ‘abnormal’ microorganisms, including *C. albicans*, *Gardnerella vaginalis* and many others. This may have introduced confounders.

We have previously discussed the history and limitations associated with serotyping/genotyping of *U. parvum* and *U. urealyticum* [23]. As a result of this, we developed a HRM PCR assay capable of detecting the four genotypes of *U. parvum* directly from clinical samples and suggested that this assay may provide valuable information relating to *U. parvum* genotype status and specific clinical conditions [23]. In the current study, using this assay, we were able to identify single *U. parvum* genotypes from 91 % of colonised samples, with a further 3 % resolved to the level of ‘mixed’ genotypes. From 74 *U. parvum* positive samples, we identified genotypes SV6 (42 %) and SV3 (36 %) as the most common, followed by SV1 (15 %). This genotype distribution is slightly different to that described by Xiao et al. [8], who reported that in 169 vaginal samples from healthy pregnant women, SV3 (63 %) was the most common, followed by SV6 (30 %), SV1 (24 %) and SV14 (4 %). This may indicate that the geographical location of the cohort influences serovar prevalence. Unfortunately, these authors did not provide any data on potential associations between genotype and adverse pregnancy outcome. However, in another sample set from the same study, Xiao et al. [8] reported a significant association between detection of *U. parvum* genotype SV6 in placental tissue and histologic chorioamnionitis, a condition that is indicative of infection-associated PTB. This corroborates our finding of a significant association between detection of *U. parvum* genotype SV6 in the vagina and PTB <37 weeks GA, in addition to our detection of this genotype in 4/6 cases of PTB <34 weeks GA.

To our knowledge, the only other study that has examined *U. parvum* genotypes present in vaginal samples is that of De Francesco et al. [46], who collected cervical, urethral, and vaginal swabs from 806 women. In contrast to our results, these authors reported that genotypes SV3 and SV14 (not separated beyond this level) and SV1 were the most common detected, representing 39 and 37 % of the 158 women who were positive for *Ureaplasma* spp. Genotype SV6 was detected in 24 % of cases and was apparently associated with a vaginal microbiota deemed ‘normal’, compared to that associated with SV3/SV14, which was linked to an absence of lactobacilli. However, it is difficult to compare these results to those from our study, as De Francesco et al. [46] did not differentiate the patient type associated with positive samples, instead compiling women from outpatients for gynaecological health care control, routine screening for pregnancy, infertility problems and those with symptoms of genital infections into one group. As such, the multiple phenotypes present make it impossible to extrapolate phenotype-specific data from this study.

Another study that examined *U. parvum* genotype distribution was by Sung et al. [47] and involved preterm neonates with bronchopulmonary dysplasia (BPD). It reported that SV3 and SV6, either alone or together, accounted for 96 % of *U. parvum* isolates detected in endotracheal and/or nasopharyngeal aspirates. However, these authors failed to find an association between genotype and the development of moderate to severe BPD.

Also of interest, and particularly relevant to the clinical use of our HRM assay, we noted that *U. parvum* genotype colonisation in our study was typically singular and in nearly all cases was stable throughout pregnancy. Although we are unable to compare this to the work of any previous studies due to omission of details relating to single/multiple genotype detection [46] or presenting multiple genotype data including *U. urealyticum* genotypes [8, 47], it is interesting to note that studies that have looked at *U. urealyticum* genotype distribution generally report that when detected, multiple genotypes are typically present [8, 47, 48]. However, this may also be a result of problems with cross-reactivity associated with PCR assays for these genotypes [8].

We did not detect a significant association between the presence of vaginal *Candida* spp. alone and PTB, a result that contradicts four previous studies [4–7] and a recent systematic review [49], although we did observe a trend towards significance. This warrants further investigation in a larger cohort of women. However, when vaginal colonisation by *U. parvum* was accompanied by *C. albicans* (the most commonly detected *Candida* spp. in our study), the odds ratio (OR) for risk of PTB increased marginally from 3.32 (*U. parvum* alone) to 3.77 (both organisms). Stratifying *U. parvum* by genotype, additional increases in risk were seen when *C. albicans* was combined with *U. parvum* genotype SV6, with the OR rising from 4.17 to 5.16. Both organisms have previously been associated with increased PTB risk [1, 5, 50], while colonisation of amniotic fluid by *C. albicans* has been linked to severe fetal injury [51–53]; however, no previous studies have looked at the effects of combined asymptomatic vaginal colonisation and pregnancy outcome, particularly regarding *U. parvum* genotype. Further research is warranted, firstly to confirm our novel findings in a larger cohort, and second to attempt to uncover what factors may be responsible for this association. If our findings are confirmed, then detection of these two organisms during the early second trimester of pregnancy could prove to be a useful indicator of women at a high risk of infection-associated PTB and therefore targets for antimicrobial therapy.

Although we have discussed the results of our study in terms of presence/absence of specific bacteria at ~21 weeks GA (primarily as this is of major relevance to allow implementation of suitable treatment regimens for prevention of PTB), our study also examined vaginal samples at two additional time points (~28 weeks and ~36 weeks GA). To the best of our knowledge, this is the first study to examine the dynamics of *Ureaplasma* spp., *Mycoplasma* spp. and *Candida* spp. throughout the second and third trimesters of pregnancy. We demonstrated that detection of all organisms (with the exception of *C. glabrata*) was very stable throughout the three sampling points, particularly so for *M. genitalium* and *U. parvum*. In addition, for *U. parvum* we also demonstrated that in nearly all cases, the genotype detected at recruitment was maintained throughout the second and third time points. These are very important findings as they demonstrate that detection of these organisms early in the second trimester of pregnancy is highly predictive of their presence both early and late in the third trimester, and provides valuable information for future diagnostic assays aiming to predict women at risk of PTB.

Of significant relevance to use of *U. parvum* detection assays in future clinical diagnostic applications, we showed near 100 % concordance between detection of *Ureaplasma* spp. in clinical samples using either culture-based or PCR methods, in contrast to some previous authors who reported disparities between the two techniques [54–56]. It has been well established that *Ureaplasma* sp. cells are fragile and in order to optimise recovery from clinical samples, special attention needs to be paid to sample collection, storage and transportation, in addition to the media used for culture [57]. Our results are most likely a reflection of our highly stringent sample collection and culture conditions, whereby swabs were immediately placed into UTM media, stored at 4 °C and cultured in 10B broth within 24 h of collection under microaerophilic conditions. This is important, as despite the increased use of molecular methods for detection of *Ureaplasma* spp., culture is likely to play a key role alongside these in future studies attempting to document virulence traits of strains associated with specific disease phenotypes.

In addition to culturing *Ureaplasma* spp. from our clinical samples, we also documented the titre of organisms present in each. We identified a small, but significant association between the titre of *U. parvum* and PTB, with an average *U. parvum* titre of 10^6^ CCU in cases of PTB vs. 10^5^ CCU for term cases. Although this may appear significant statistically, it is unlikely to be clinically relevant. We used eight 10-fold serial dilutions of 10B broth culture for quantitation and expressed our titres in colour changing units (CCU), a technique that has been widely used in *Ureaplasma* sp. culture [58–61]. A limitation of the CCU method is that although it is far more suited to quantitation of large sample numbers, it really is semi-quantitative in that the titre values obtained actually represent a range rather than hard values. For instance, 10^6^ CCU may actually be 1,000,000–9,000,000 cells. As a result, we believe clinical significance would have only been justified in the case of a difference of two dilution series, as opposed to a single series, such as we observed. Standard solid agar plate quantitation methods are not well suited to *Ureaplasma* spp. due to the tiny colony size and associated quantitation difficulties (a microscope with ~ 10–40× magnification is required). Despite this, Abele-Horn et al. [35] used this method for quantitation and following multivariate analysis of 172 *Ureaplasma*-colonised and 123 non-colonised pregnant women, they reported that *Ureaplasma* sp. vaginal colonisation was an independent risk factor for PTB at 10^5^ CFU/mL and for chorioamnionitis at both 10^4^–10^5^ and 10^5^ CFU/mL. Lower colonisation levels had no adverse effects on pregnancy outcome [35]. Regardless of the method used though, it could be argued that accurate quantitation of any bacteria from a swab-collected sample is confounded by the manner of sample collection, which is likely to have a large effect on the results.

Although our sample size was too small to accurately document any association between detection of vaginal *M. genitalium* and PTB, we observed a trend towards significance, with detection rates of 12 and 2 % at recruitment in women delivering preterm and term, respectively. This result is particularly interesting in that it conflicts with the findings of four previous studies, all of which found no association between vaginal *M. genitalium* and PTB; interestingly, they reported detection rates (0.7–8 %) somewhat lower than those reported here [62–65]. One of these was by Labbe et al. [65] who reported rates of 8 % in preterm deliveries (16/183) and 6 % in women delivering at term (36/564) in a population of women from a developing country, many of whom also screened positive for other known sexually transmitted infections, including human immunodeficiency virus (HIV). In comparison, Hitti et al. [66], reported detection rates of only 4 % (29/661) and 2 % (12/667) in Peruvian women who delivered preterm and term, respectively. This finding was significant, accompanied by an odds ratio of 2.5 (95 % CI: 1.2–5.0). Of particular interest, both Oakeshott et al. [63] and Hitti et al. [66] documented that vaginal *M. genitalium* was more commonly observed in women of a younger maternal age (<20 years and mean 21.2 years, respectively). This was also true in our cohort, where the mean age of women in which vaginal *M. genitalium* colonisation was detected was 24.3 years (in comparison to the overall cohort mean age of 30), and perhaps suggests that maternal age needs to be taken into account when assessing microbiological-associated PTB risk.

Many previous studies that have examined associations between vaginal bacterial colonisation and PTB have not considered the effect of lifestyle factors on the species present. By administering questionnaires to our participants at each sampling point (asking about such lifestyle factors as smoking before/during pregnancy and the amount of sexual intercourse during pregnancy), we sought to establish if there was a relationship between these factors and organism presence. For both *Ureaplasma* spp. and *Mycoplasma* spp., we observed significantly higher detection rates in women who reported having sexual intercourse greater than or equal to 3 times per week during the course of their pregnancy. Considering that both of these organisms are known colonisers of both the female and male genital tracts [67], and are also considered sexually-transmitted infections in certain circumstances [20], this result is not surprising. However, of particular interest, we also observed associations between women who either smoked prior to or during their pregnancy and increased detection rates of *Ureaplasma, Mycoplasma* and/or *Candida* spp. Of statistical significance, higher detection rates of *Ureaplasma* spp. (37 vs. 17 %) and *Mycoplasma* spp. (44 vs. 24 %) were observed in women who smoked prior to their pregnancy and for *Candida* spp., colonisation rates were significantly higher in women who continued to smoke throughout their pregnancy (18 vs. 7 %). A similar phenomenon has been reported previously in relation to smoking and vaginal microbiology in the case of human papillomavirus (HPV) infection [68] and also for bacterial vaginosis [15]. Although the association between BV and smoking may help to explain the association between *Ureaplasma* spp. and *Mycoplasma* spp., both of which have been previously reported as BV-associated agents [3], *Candida* spp. are not typically associated with BV. In addition, whereas it has been reported that the anti-estrogenic effect of smoking [69] may predispose a woman to BV [16], it has been previously documented that *Candida* spp. are more prevalent during pregnancy as a result of the increased amounts of estrogen present [70]. One possible explanation could be that benzo[a]pyrene, found in trace amounts in the vaginal secretions of smokers, has been shown to significantly induce *Lactobacillus* spp. prophages [71]. This would result in a decrease in numbers of vaginal *Lactobacillus* spp. due to cell lysis from lytic phages, potentially providing a more favourable environment for *Candida* spp. and especially BV-associated organisms, as *Lactobacillus* spp. are critical for maintenance of an acidic vaginal pH [72]. Detailed metagenomics studies would be required to address this hypothesis.

## Conclusions

Although many previous studies have shown associations between *Ureaplasma* spp. and PTB, our study has demonstrated the importance of detecting *U. parvum* and *U. urealyticum* as individual species, and for *U. parvum*, genotype, when investigating the relationship between vaginal colonisation and PTB risk. We found that vaginal *U. parvum* colonisation was associated with increased risk of spontaneous PTB, and that detection of *U. parvum* genotype SV6 was of even higher significance, especially when combined with the presence of *C. albicans*. This result now needs to be confirmed in a larger cohort of women, preferably in both similar and different ethnic populations, in order to evaluate its use as an early detection biomarker for risk of PTB. Our finding that colonisation status is preserved across pregnancy is clinically important, suggesting that for *Ureaplasma*, *Mycoplasma* and *Candida* spp., a single sample taken in pregnancy has diagnostic and prognostic utility. The cause and effect relationships between colonisation with *U. parvum* genotype SV6, *C. albicans* and preterm labour, however, are unproven. Further work is currently underway to attempt to identify potential virulence factors at the genome level, in addition to describing the relationship between vaginal colonisation and intraamniotic infection with specific organisms in order to illuminate a plausible mechanism. Lastly, our finding that smoking either prior to pregnancy or during pregnancy is significantly associated with *Ureaplasma*, *Mycoplasma* and *Candida* spp. vaginal colonisation status offers additional insight into relationships between lifestyle and pregnancy risk. Larger studies are needed to confirm these findings in cohorts of past and current smokers.
